# Angiogenesis after administration of basic fibroblast growth factor induces proliferation and differentiation of mesenchymal stem cells in elastic perichondrium in an in vivo model: mini review of three sequential republication-abridged reports

**DOI:** 10.1186/s11658-018-0113-1

**Published:** 2018-10-05

**Authors:** Toru Miyanaga, Yoshimichi Ueda, Aiko Miyanaga, Mikio Yagishita, Naoko Hama

**Affiliations:** 1 Department of Plastic and Reconstructive Surgery, Kanazawa Medical University, 1-1 Daigaku, Uchinada-machi, Kahoku-gun, Ishikawa 9200293 Japan; 2 Department of Pathology, Kanazawa Medical University, 1-1 Daigaku, Uchinada-machi, Kahoku-gun, Ishikawa 9200293 Japan; 3 Department of Nursing, Kanazawa Medical University, 1-1 Daigaku, Uchinada-machi, Kahoku-gun, Ishikawa 9200293 Japan; 4 Kanazawa Medical University Hospital, 1-1 Daigaku, Uchinada-machi, Kahoku-gun, Ishikawa 9200293 Japan

**Keywords:** Angiogenesis, Basic fibroblast growth factor, Differentiation, Elastic cartilage, In vivo model, Mesenchymal stem cell, Progenitor cell, Proliferation

## Abstract

To date, studies on mesenchymal tissue stem cells (MSCs) in the perichondrium have focused on in vitro analysis, and the dynamics of cartilage regeneration from the perichondrium in vivo remain largely unknown. We have attempted to apply cell and tissue engineering methodology for ear reconstruction using cultured chondrocytes. We hypothesized that by inducing angiogenesis with basic fibroblast growth factor (bFGF), MSCs or cartilage precursor cells would proliferate and differentiate into cartilage in vivo and that the regenerated cartilage would maintain its morphology over an extended period. As a result of a single administration of bFGF to the perichondrium, cartilage tissue formed and proliferated while maintaining its morphology for at least 3 months. By day 3 post bFGF treatment, inflammatory cells, primarily comprising mononuclear cells, migrated to the perichondrial region, and the proliferation of matrix metalloproteinase 1 positive cells peaked. During week 1, the perichondrium thickened and proliferation of vascular endothelial cells was noted, along with an increase in the number of CD44-positive and CD90-positive cartilage MSCs/progenitor cells. Neocartilage was formed after 2 weeks, and hypertrophied mature cartilage was formed and maintained after 3 months. Proliferation of the perichondrium and cartilage was bFGF concentration-dependent and was inhibited by neutralizing antibodies. Angiogenesis induction by bFGF was blocked by the administration of an angiogenesis inhibitor, preventing perichondrium proliferation and neocartilage formation. These results suggested that angiogenesis may be important for the induction and differentiation of MSCs/cartilage precursor cells in vivo, and that morphological changes, once occurring, are maintained.

## Introduction

Ear reconstruction using cell and tissue engineering methods involving cultured chondrocytes has been attempted. Although cultured mature cells possess a high ability to form cartilage tissue, there are defects in long-term maintenance because of a low capacity for regeneration [1]. Kobayashi et al. succeeded in purifying mature cartilage tissue by identifying mesenchymal stem cells (MSCs) and progenitor cells among human auricular cartilage cells, and in culturing the cells [2]. Using a similar method, Kagimoto et al. injected cultured human and monkey perichondrial cells into immunodeficient mice and confirmed that mature cartilage tissue is not absorbed by 3 months after production [3]. They reported that the self-renewal ability of MSCs makes it possible to maintain long-term morphological function. In addition, Takebe et al. revealed that vascular endothelial cells are important for MSC differentiation into cartilaginous tissue in the perichondrium, and demonstrated in vitro that self-regeneration of MSCs occurred as a result of vascular endothelial cell formation [4]. To date, studies on MSCs in the perichondrium have focused on in vitro analyses, with the details of cartilage regeneration from the perichondrium in vivo remaining largely undefined [2, 5].

We hypothesized that by inducing angiogenesis, MSCs/cartilage precursor cells would proliferate and differentiate into cartilage in vivo and that the regenerated cartilage would maintain its morphology over an extended period of time. Accordingly, we conducted an experimental investigation using basic fibroblast growth factor (bFGF) to induce angiogenesis. The growth factor bFGF promotes the proliferation, differentiation, and migration of various cells; exhibits strong angiogenic action [6]; and has been studied as a major component in the wound healing process [7]. The specific aims of the current study were to determine whether bFGF would induce cartilage proliferation in vivo in the rabbit elastic perichondrium, and to investigate the involvement of MSCs and angiogenesis in this model system.

## Materials and methods

### Animal model

All experimental protocols involving animals and their tissues were approved by the Ethics Committee of Kanazawa Medical University School of Medicine. Japanese white male rabbits were purchased from Sankyo Labo Service Corporation (Toyama, Japan); 57 rabbits (aged 14–16 weeks; weighing 2.5–3.5 kg) were housed in individual cages under a 12 h/12 h light/dark cycle with free access to food and water. All the rabbits were anesthetized with pentobarbital (25 mg/kg) through ear marginal vein injection prior to the surgical procedure**.** The details of the surgical procedures have been mentioned in each experiment section. At the end of the experiment, the rabbits were euthanized. After euthanasia, the experimental areas and a nonexperimental area of the rabbits’ ears were excised, fixed in 10% buffered formalin, and embedded in paraffin.

### Histological and immunohistochemical analyses

Paraffin-embedded sections were subjected to hematoxylin and eosin (HE) staining using standard procedures. Immunohistochemical staining was performed using the streptavidin-biotin-peroxidase-complex method (Histofine SAB-PO kit, Nichirei Co., Tokyo Japan). Tissue sections were deparaffinized and rehydrated prior to immunostaining. Tissues sections were treated with proteinase K (20 mg/ml; Dako Cytomation, Carpinteria, CA, USA) for 10 min at room temperature for antigen activation, except for Ki67 staining in which antigen activation was performed using tris-acetate-EDTA buffer (Target Retrieval Solution, Dako Cytomation, Carpinteria, CA, USA) for 4 h at 37 °C, endogenous peroxidase activity was quenched with 3% hydrogen peroxide in methanol, and tissue sections were blocked with 10% serum-free protein block. Primary peroxidase-conjugated antibodies included anti-type 1 collagen (0.25 μg/ml; Daiichi Fine Chemical, Toyama, Japan) for the primary extracellular-matrix component of the perichondrium, anti-type 2 collagen (2.5 μg/ml; Daiichi Fine Chemical) for the primary extracellular-matrix component of chondrium, anti-CD44 (1 μg/ml; Eptimics, Burlingame, CA, USA), anti-CD34 (2 μg/ml; Leica, UK) as a negative marker, anti-MMP-1 (1.25 μg/ml; Daiichi Fine Chemical, Toyama, Japan), anti-CD31 antibody JC/70A (5 μg/ml; Novus Biologicals, Littleton, CO, USA), and anti-Ki67 antibody MIB-1 (10 μg/ml; Dako Cytomation, Carpinteria, CA, USA). The experimental tissue sections were incubated with the primary antibodies at 4 °C overnight. For colorimetric detection, 3,3′-diaminobenzidine (DAB) was used as the peroxidase substrate. The specimens were counterstained with Mayer’s hematoxylin.

### Thickness of cartilage and perichondrium

Immunohistochemical staining of type 1 and 2 collagen expression was performed for evaluation of the perichondrium and chondrogenesis, respectively. Thickness of cartilage and perichondrium was measured using Image J (version 1.45) image analysis software (National Institutes of Health, Bethesda, MD, USA).

### Temporal quantification of MMP-1-, CD31-, Ki67-, and CD90-positive cells

Cells positive for MMP-1, CD31, Ki67, and CD90 were counted in randomly chosen microscopic visual fields (× 200) of the perichondrium layer and the subcutaneous layer close to the perichondrium (a width of 200 μm). Vessels with a diameter less than 50 μm were counted to quantitate the level of neovascularization.

### Effect of a single administration of bFGF

In brief, 18 animals received 0.1 ml of recombinant human bFGF (100 μg/ml, Kaken, Japan) by subcutaneous injection into the perichondrium tissue of the auricular region using insulin syringes (29G × 13 mm, Terumo, Tokyo, Japan). Each rabbit received injections in four locations. At 1, 3, 7, and 14 days, and at 1 month and 3 months post-injection, three rabbits per group were euthanized. Three microscopic visual fields were randomly chosen for 12 tissue specimens from each of three rabbits (*n* = 36). In a preliminary study, there was no histological difference between control saline-injected (0.1 ml) areas and noninjected areas at each time point; therefore, the control groups throughout the remainder of the study were noninjected areas. The thickness of cartilage was measured and cells positive for MMP-1, CD31, Ki67, and CD90 were counted.

### Effect of bFGF concentration on auricular chondrogenesis

To determine the importance of bFGF concentration, 0.1 ml of bFGF in normal saline at concentrations of 1, 5, 10, 25, 50, and 100 μg/ml was injected subcutaneously into the perichondrium in the auricular region of the rabbits. The noninjected areas were treated as the control areas. Three rabbits were used, and each rabbit received injections in six locations (each bFGF concentration for each rabbit). At 1 month post-injection, the injected region was excised. Each section was stained with HE. The thickness of new cartilage and the origin of cartilage production were evaluated at five points (*n* = 15) in randomly chosen microscopic fields. The rate of new cartilage formation was calculated using the following equation:

$$ \mathrm{Cartilage}\kern0.5em \mathrm{Neogenesis}\kern0.5em \mathrm{Rate}\kern0.5em =\kern0.5em \frac{\mathrm{New}\kern0.5em \mathrm{Cartilage}\kern0.5em \mathrm{Thickness}}{\left(\mathrm{Original}\kern0.5em \mathrm{Cartilage}\kern0.5em \mathrm{Thickness}\kern0.5em +\kern0.5em \mathrm{New}\kern0.5em \mathrm{Cartilage}\kern0.5em \mathrm{Thickness}\right)} $$

### Blocking bFGF stimulation using a neutralizing antibody

Fifteen rabbits were randomly divided into five groups of three rabbits each. bFGF in normal saline (0.1 ml; 5 μg/ml) was injected subcutaneously into the perichondrium in the auricular region of each rabbit, followed by the injection of 0.25 ml of an anti-bFGF neutralizing monoclonal antibody (1 mg/ml, R&D systems, USA) into the same region at the various time points indicated. The groups of animals were injected with the neutralizing antibody as follows: group 1 (G1), immediately after the bFGF injection; group 2 (G2), immediately and 1 week post bFGF-injection; group 3 (G3), immediately and 2 weeks post bFGF-injection; group 4 (G4), 1 week post bFGF-injection; and group 5 (G5), 2 weeks post bFGF-injection. The injected area and the noninjected control areas were excised 1 month post bFGF-injection, fixed in 10% buffered formalin, and embedded in paraffin. The areas not injected with the neutralizing antibody after bFGF-injection were the control areas. Sections were HE-stained, and the neogenesis cartilage rate was calculated.

### MMP inhibition

bFGF (0.1 ml, 100 μg/ml) was injected subcutaneously into the perichondrium in the auricular region of ten rabbits. At 24-h after the bFGF treatment, 0.10 ml of 6.25 mM MMP inhibitor (Wako, Osaka, Japan), or the DMSO carrier alone (as a negative control), was locally injected into each ear. The dosing and timing for the MMP treatment were based on preliminary experiments. Each rabbit received injections in four locations (2 experimental and 2 control regions). Each of 5 rabbits was euthanized at 1-week and 2-weeks post MMP treatment (*n* = 10 per time point). The tissue was sectioned on a microtome and stained with HE. The thickness of new cartilage was measured at the center of the region of injection, and cells positive for CD31 were counted in five randomly chosen microscopic visual fields (× 200).

### VEGF neutralization

Eleven rabbits were treated with 0.10-ml subcutaneous injections of bFGF (100 μg/ml) in the perichondrium in the auricular region. Based on preliminary experiments, 0.10 ml of localized treatment of VEGF-neutralizing antibody (25 mg/ml; Chugai, Tokyo, Japan), or normal saline as a negative control, was administered by three subcutaneous injections into the same region at 30 min, 24 h, and 48 h post bFGF treatment. Each rabbit received injections in four locations (2 experimental and 2 control regions). Five rabbits were euthanized 1 week later (*n* = 10 per time point), and six rabbits were euthanized at 2 weeks after the initial bFGF stimulation (*n* = 12 per time point). Measurement of the thickness of new cartilage and the number of CD31-positive cells was performed as described above.

### Statistical analysis

Experiment of a single administration of FGF: two-way analysis of variance (ANOVA) was performed to compare the means of two or more groups for the objective study period. A *t*-test or one-way ANOVA was also conducted to compare the means of two or more groups on each day of measurement. Scheffe’s method of multiple comparisons was used as a post hoc analysis to evaluate the differences in all combinations of two individual groups for data from each day of measurement using the StatView 5.0 software (Cary, NC).

Experiment of bFGF concentration and blocking bFGF stimulation using a neutralizing antibody: nonparametric testing for variance by Kruskal–Wallis analysis was performed to determine significance in cartilage neogenesis rate using StatView software version 5.0 (Cary, NC). Post hoc comparisons by Scheffe’s test and Tukey HSD test were performed when necessary.

Experiment of MMP inhibition and VEGF neutralization: Student’s t-tests were used to assess for significance using SPSS v. 20 (IBM Corp, Japan).

Results were considered statistically significant if *p* < 0.05.

## Results and discussion

### BFGF-induced proliferation and differentiation of MSCs or cartilage precursor cells in ear cartilage in vivo *model*

**Fig. 1:**
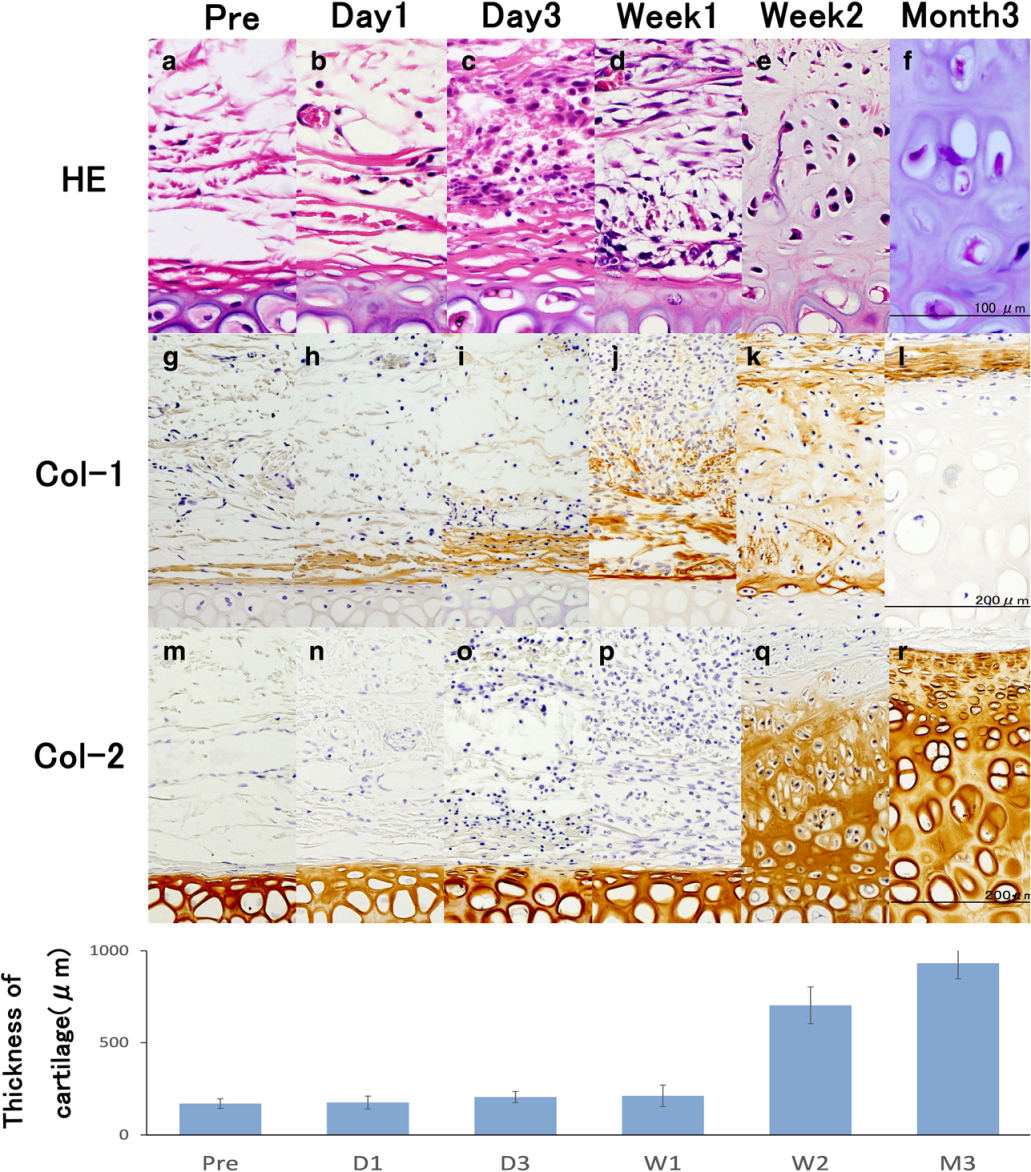
Histological and biochemical evaluation of the auricular cartilage proliferation model following bFGF administration. **a–f**: Histological staining of rabbit perichondrium tissue with hematoxylin and eosin (HE), **g–l**: biochemical evaluation of rabbit perichondrium tissue immunostained with anti-type-1 collagen peroxidase-conjugated antibody, **m–r**: biochemical evaluation of rabbit perichondrium tissue immunostained with anti-type-2 collagen peroxidase-conjugated antibody. **a**, **g**, **m**: pre-injection of bFGF; **b**, **h**, **n**: day 1 post bFGF-injection; **c**, **i**, **o**: day 3 post bFGF-injection; **d**, **j**, **p**: week 1 post bFGF-injection; **e**, **k**, **q**: week 2 post bFGF-injection; **f**, **l**, **r**: month 3 post bFGF-injection. Abbreviations: Pre, pre-injection of bFGF (control); D1, day 1 post bFGF-injection; D3, day 3 post bFGF-injection; W1, week 1 post bFGF-injection; W2, week 2 post bFGF-injection; M3, month 3 post bFGF-injection. **a** There were a few fibroblasts and endothelial cells in subcutaneous tissues. **g** Pericartilage cells shaped like fibroblasts in pericartilage located in the outer layer of cartilage that was immunostained for type 1 collagen. **m** Cartilage was immunohistochemically stained for type 2 collagen. One to 3 days after injection of a bFGF, mononuclear cell infiltrates mostly comprised macrophages (**b**, **c**), and volume expansion of the perichondrium layer was observed in the superficial perichondrium and the subcutaneous layer proximal to the perichondrium (**h**, **i**), but no changes in the cartilage were observed (**n**, **o**). One week after injection of bFGF, proliferation of the perichondrium cells with spindle and spider shapes (**d**) and thick extracellular matrix with type 1 collagen fiber were observed (**j**), but the cartilage was not changed (**p**). Two weeks after injection of bFGF, the superficial perichondrium layer and the deep neo-chondrium layer with neocartilage cells differentiated from the pericartilage cells (**e**), extracellular matrix with decreased type 1 collagen (**j**), and type 2 collagen fiber were observed (**q**). Three months after the injection, there were neocartilage cells with hypertrophy of the cytoplasm, and the cartilage was similar to mature tissue (**f**), whereas the extracellular matrix with type 1 collagen was limited in the outer layer of the neo-chondrium and abundant extracellular matrix with type 2 collagen was observed (**r**). The graph shows the mean thickness of the cartilage over the duration of the study. The differences in the thickness of cartilage between 1 week and 2 weeks were statistically significant, with *P* < 0.0001. “Reprinted with permission from (Yagishita, M. 2013. Involvement of mesenchymal tissue stem cells and aquaporin 1 in rabbit auricular cartilage regeneration from perichondrium with full reference. Journal of Kanazawa Medical University, 38, 43–52.). Copyright (2013) publication administration of Kanazawa Medical University”

Representative findings from the evaluation of the in vivo rabbit model with a single bFGF treatment are shown in Fig. 1. One to 3 days after the injection of 10 μg of bFGF (100 μg/ml), infiltration of mononuclear cells, comprising mostly macrophages, and an expansion in the volume of the perichondrium layer in the superficial perichondrium and the subcutaneous layer near the perichondrium were observed. One week after the injection, proliferation of perichondrium cells with spindle and spider shapes, and extracellular matrix with type 1 collagen fibers were observed. Two weeks after the injection, the superficial perichondrium layer and the deep neo-chondrium layer with neocartilage cells were differentiated from pericartilage cells. Additionally, by 2 weeks, type 2 collagen fibers in the extracellular matrix were observed. From one to 3 months after the injection, neocartilage cells with hypertrophy of the cytoplasm and abundant extracellular matrix were present. The new cartilage was morphologically similar to mature tissue.

The average thickness of the cartilage stained with an anti-type 2 collagen antibody (Fig. 1) was 171 ± 26 μm for the control, 177 ± 35 μm at 1 day, 206 ± 30 μm at 3 days, 213 ± 58 μm at 1 week, 703 ± 100 μm at 2 weeks, 835 ± 84 μm at 1 month (not shown in Fig. 1), and 933 ± 18.8 μm at 3 months. The differences in the thickness of cartilage between 1 week and 2 weeks were statistically significant (*P* < 0.0001).

**Fig. 2:**
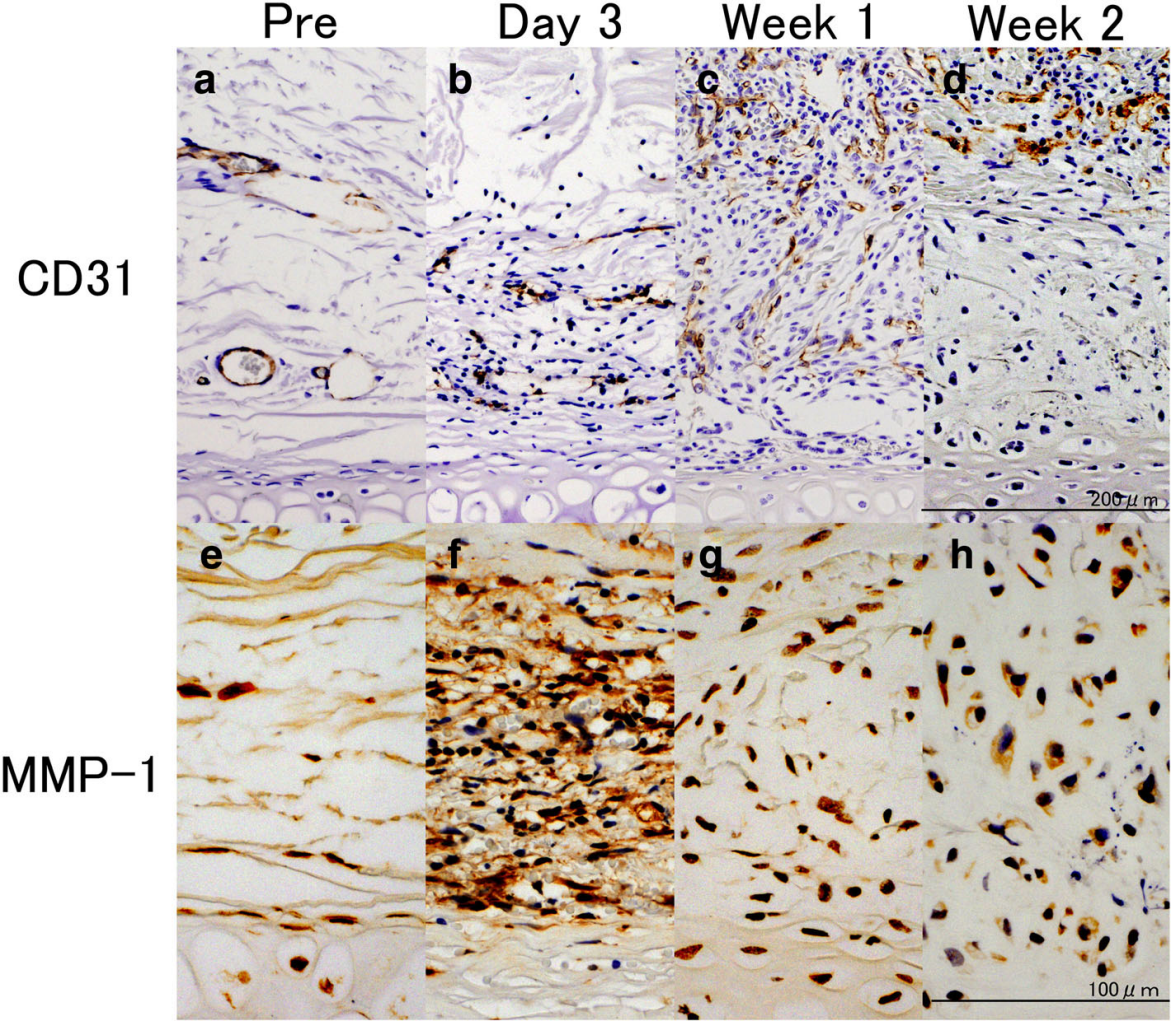
Endothelial (CD31-positive) cells and MMP-1-positive cells. **a–d**: Biochemical evaluation of rabbit perichondrium tissue immunostained with anti-CD31 peroxidase-conjugated antibody, **e–h**: biochemical evaluation of rabbit perichondrium tissue immunostained with anti-MMP-1 peroxidase-conjugated antibody. **a**, **e**: Pre-injection of bFGF; **b**, **f**: day 3 post bFGF-injection; **c**, **g**: week 1 post bFGF-injection; **d**, **h**: week 2 post bFGF-injection. **a** CD31-positive cells were scarcely observed around the perichondrium layer in the noninjected group. **b** Mononuclear cells and small vascular endothelial cells were observed on day 3. **c** Proliferation of vascular endothelial cells was noted and the vessels extended vertically into the perichondrial region on Week 1. Vascular endothelial cells were not observed in the deep layer of the perichondrium, but were located in the superficial layer on Week 2. **e** A small number of MMP-1-positive cells in the perichondrium was observed in the noninjected group. **f** A large number of mononuclear cells and pericartilage cells stained positive for MMP-1 in the perichondrium and the outer layer of the perichondrium. **g**, **h** Only mononuclear cells stained positive for MMP-1 in the superficial layer of the perichondrium during Week 1 and 2. Reprinted with permission from (Yagishita, M. 2013. Involvement of mesenchymal tissue stem cells and aquaporin 1 in rabbit auricular cartilage regeneration from perichondrium with full reference. Journal of Kanazawa Medical University, 38, 43–52.). Copyright (2013) publication administration of Kanazawa Medical University. “Reprinted with permission from (Yagishita, M. 2013. Involvement of mesenchymal tissue stem cells and aquaporin 1 in rabbit auricular cartilage regeneration from perichondrium with full reference. Journal of Kanazawa Medical University, 38, 43–52.). Copyright (2013) publication administration of Kanazawa Medical University”

CD31-positive cells were seldom observed around the perichondrium layer in the noninjected group (Fig. 2). Mononuclear cells and small vascular endothelial cells were observed on day 3 following the administration of bFGF. During the first week, proliferation of vascular endothelial cells was noted and the vessels extended vertically into the perichondrial region. Vascular endothelial cells were not observed in the deep layer of perichondrium, but were located in the superficial layer during week 2.

As shown in Fig. 2, a small number of MMP-1-positive cells in the perichondrium was observed in the noninjected control group. During day 3, a large number of mononuclear cells and pericartilage cells stained positive for MMP-1 in the perichondrium and the outer layer of perichondrium. Only mononuclear cells stained positive for MMP-1 in the superficial layer of the perichondrium during the first week following bFGF treatment.

CD44 and CD90 are markers for MSCs of auricular cartilage, whereas CD34 is not and served as a negative control. The protein Ki-67 is a cellular marker for proliferation.

**Fig. 3:**
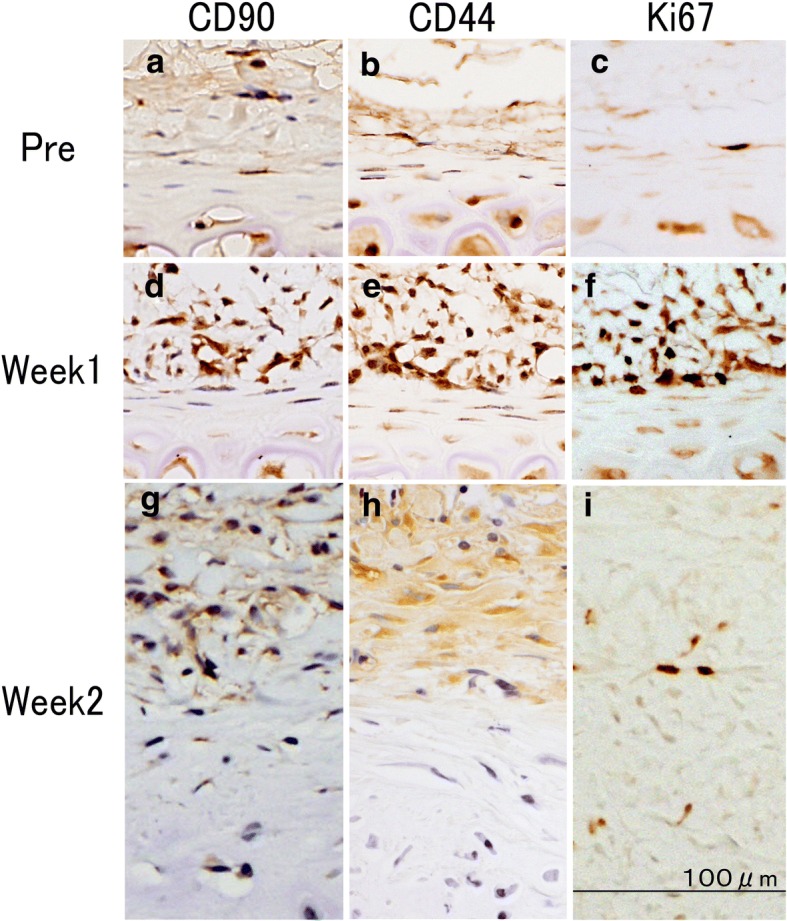
Identification of mesenchymal tissue stem cells (MSCs) in the cartilage proliferation model. CD44 and CD90 are markers for MSCs of auricular cartilage and Ki-67 protein is a cellular marker for proliferation. **a**, **d**, **g**: Biochemical evaluation of rabbit perichondrium tissue immunostained with anti-CD90 peroxidase-conjugated antibody; **b**, **e**, **h**: biochemical evaluation of rabbit perichondrium tissue immunostained with anti-CD44 peroxidase-conjugated antibody; **c**, **f**, **i**: biochemical evaluation of rabbit perichondrium tissue immunostained with anti-Ki67 peroxidase-conjugated antibody. **a**-**c**: Pre-injection of bFGF; **d**-**f**: week 1 post bFGF-injection; **g**-**i**: week 2 post bFGF-injection. A small number of CD44-positive and CD90-positive cells was observed in the noninjected group (**a**, **b**), but a large number of CD44-positive and CD90-positive cells was observed 1 week after the injection of bFGF. Most of spindle- and spider-shaped cells in the perichondrium layer stained positive for CD44 and CD90 (**d**, **e**). These positive cells decreased in number as the perichondrium layer thinned 2 weeks after the injection (**g**, **h**). Ki-67-positive cells were scarcely observed in noninjected controls or one day after injection (**c**). During week 1, Ki67-positive perichondrial cells were observed in the outer layer from the perichondrial inner-layer/outer-layer boundary (**f**). Only perichondrial cells stained positive for the Ki-67 marker in the superficial layer of the perichondrium during week 2 (**i**). “Reprinted with permission from (Yagishita, M. 2013. Involvement of mesenchymal tissue stem cells and aquaporin 1 in rabbit auricular cartilage regeneration from perichondrium with full reference. Journal of Kanazawa Medical University, 38, 43–52.). Copyright (2013) publication administration of Kanazawa Medical University”

As shown in Fig. 3, a small number of CD44-positive and CD90-positive cells was observed in the noninjected group, but a large number of CD44-positive and CD90-positive cells was observed 1 week after the injection of bFGF. Almost all the observed spindle- and spider-shaped cells in the perichondrium layer stained positive for CD44 and CD90. These positively stained cells decreased in number 2 weeks after the injection. No CD34-positive cells were detected in any of the experimental groups. Ki-67-positive cells were scarcely observed in noninjected controls at 1 day after the injection. During week 1, Ki67-positive perichondrial cells were observed in the outer region of the perichondrial inner-layer/outer-layer boundary. In the superficial layer of perichondrium during week 2, only perichondrial cells stained positive for Ki-67.

**Fig. 4:**
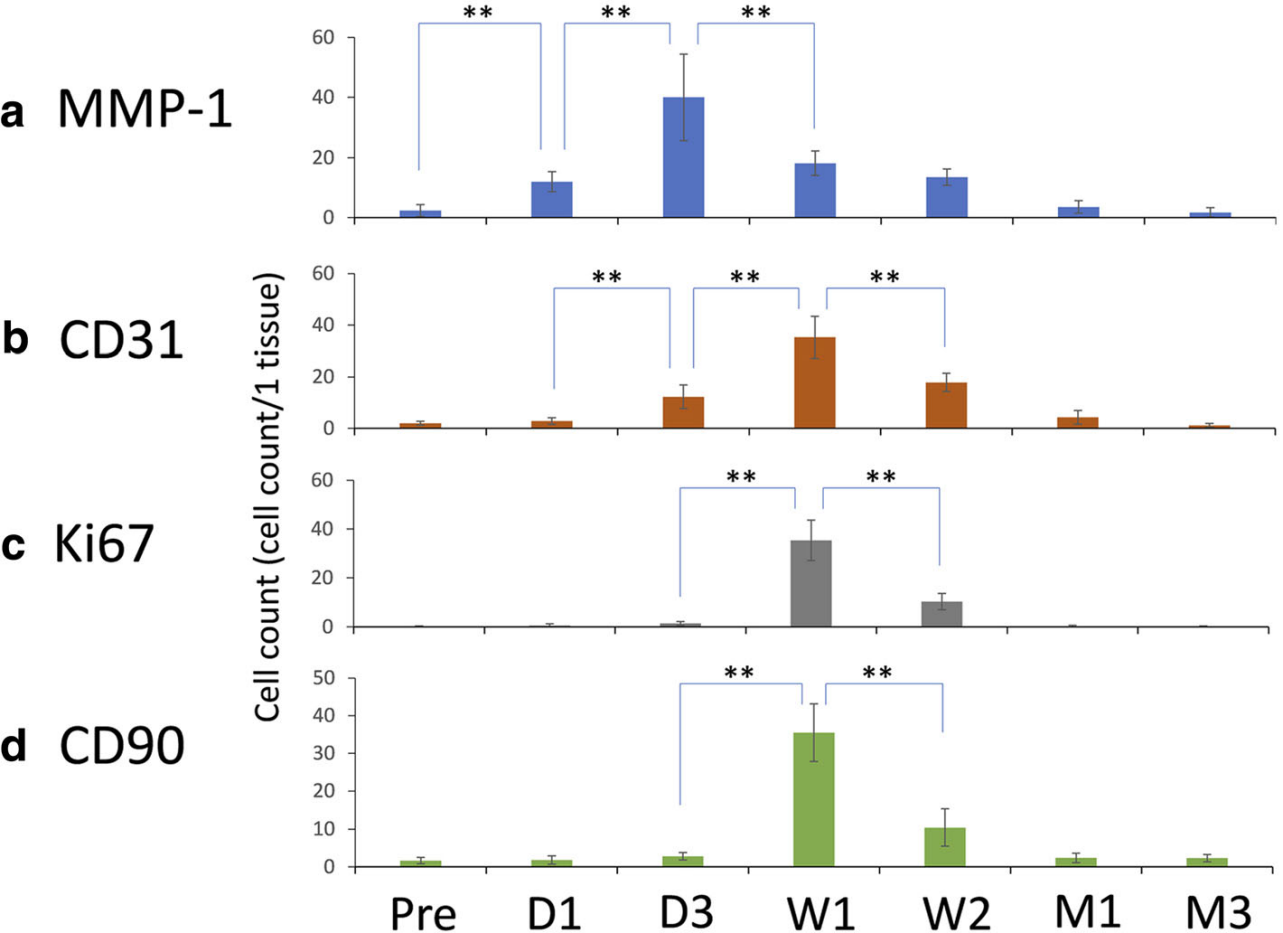
Temporal quantification of MMP-1-, CD31-, Ki67-, and CD90-positive cells in perichondrium tissue. Abbreviations: Pre, pre-injection of bFGF (control); D1, day 1 post bFGF injection; D3, day 3 post bFGF injection; W1, week 1 post bFGF injection; W2, week 2 post bFGF injection; M1, month 1 post bFGF injection; M3, month 3 post bFGF injection. **a** Immunohistochemical staining with an anti-MMP-1 antibody was performed to evaluate type-1 collagenolytic factor. The graph shows the mean numbers of MMP-1-positive cells over the duration of the study. The differences in the numbers of MMP-1-positive cells comparing the controls to 1 day, 1 day to 3 days, and 3 days to 1 week were statistically significant. **P < 0.0001. **b** Immunohistochemical staining with an anti-CD31 antibody was performed to evaluate angiogenesis. The graph shows the mean numbers of CD31-positive cells over the duration of the study. The differences in the numbers of CD31-positive cells comparing 1 day to 3 days, 3 days to 1 week, and 1 week to 2 weeks were statistically significant. **P < 0.0001. **c** Immunohistochemical staining with the anti-Ki67 antibody was performed to evaluate proliferation. The graph shows the mean numbers of Ki67-positive cells over the duration of the study. The differences in the counts of Ki67-positive cells comparing 3 days to 1 week, and 1 week to 2 weeks were statistically significant. **P < 0.0001. **d** Immunohistochemical staining using an anti-CD90 antibody was conducted to evaluate MSCs. The graph shows the mean numbers of CD90-positive cells over the duration of the study. The differences in the numbers of CD90-positive cells comparing 3 days to 1 week, and 1 week to 2 weeks were statistically significant. **P < 0.0001. “Reprinted with permission from (Yagishita, M. 2013. Involvement of mesenchymal tissue stem cells and aquaporin 1 in rabbit auricular cartilage regeneration from perichondrium with full reference. Journal of Kanazawa Medical University, 38, 43–52.). Copyright (2013) publication administration of Kanazawa Medical University”

Immunohistochemical staining with the anti-MMP-1 antibody was performed to evaluate type 1 collagenolytic activity. The average number of positive cells per microscopic visual field was 2.4 ± 2.0 for the control, 12.0 ± 3.3 at 1 day, 40.1 ± 14.4 at 3 days, 18.2 ± 4.1 at 1 week, 13.5 ± 2.7 at 2 weeks, 3.6 ± 2.1 at 1 month, and 1.7 ± 1.6 at 3 months. The differences in the number of MMP-1-positive cells from the control to 1 day, 1 day to 3 days, and 3 days to 1 week were statistically significant (*P* < 0.0001) (Fig. 4a). Immunohistochemical staining with the anti-CD31 antibody was performed to evaluate angiogenesis. The average number of positive cells per visual field was 2.0 ± 0.8 for the control, 2.9 ± 1.3 at 1 day, 12.3 ± 4.6 at 3 days, 35.3 ± 8.2 at 1 week, 17.8 ± 3.6 at 2 weeks, 4.3 ± 2.7 at 1 month, and 1.2 ± 0.7 at 3 months. The differences in the number of CD31-positive cells from 1 day to 3 days, 3 days to 1 week, and 1 week to 2 weeks were statistically significant (*P* < 0.0001) (Fig. 4b). Immunohistochemical staining with the anti-Ki67 antibody was performed to evaluate proliferation. The average number of positive cells per visual field was 0.1 ± 0.3 for the control, 0.6 ± 0.7 at 1 day, 1.4 ± 0.8 at 3 days, 35.3 ± 8.3 at 1 week, 10.4 ± 3.3 at 2 weeks, 0.2 ± 0.4 at 1 month, and 0.1 ± 0.4 at 3 months. The differences in the number of Ki67-positive cells from 3 days to 1 week, and 1 week to 2 weeks were statistically significant (*P* < 0.0001) (Fig. 4c). Immunohistochemical staining using the anti-CD90 antibody was conducted to evaluate MSCs. The average numbers of positive cells were 1.6 ± 0.8 for the control, 1.8 ± 1.1 at 1 day, 2.8 ± 1.0 at 3 days, 35.5 ± 7.7 at 1 week, 10.4 ± 3.3 at 2 weeks, 2.4 ± 1.2 at 1 month, and 2.3 ± 1.0 at 3 months. The differences in the number of CD90-positive cells from 3 days to 1 week, and from 1 week to 2 weeks were statistically significant (*P* < 0.0001) (Fig. 4d).

A single administration of bFGF to the perichondrium resulted in the proliferation of precursor cells, and cartilage tissue formation with prolonged maintenance of cartilage morphology for at least 3 months. Inflammatory cells comprising mononuclear cells migrated into the treatment site by day 3 following administration of bFGF; moreover, by this time the proliferation of MMP1-positive cells peaked. During week 1, thickening of the perichondrium occurred and proliferation of vascular endothelial cells in the perichondrial region was observed. The bFGF treatment stimulated CD44-positive and CD90-positive cartilage MSCs or progenitor cells in the perichondrium to proliferate. Subsequently, neocartilage was formed after 2 weeks, and after 3 months hypertrophied mature cartilage was formed and maintained.

It has been reported that bFGF is a growth factor for fibroblasts present in bovine-brain extract [8]; it promotes the proliferation, differentiation, and migration of various cells and is a growth factor with strong angiogenic action [6]. The growth-promoting effect of administration of bFGF on mature chondrocytes has been previously reported, but the effects on perichondrial tissue or perichondrial cells have not been elucidated. Moreover, it is unknown which cells in the perichondrium are involved in proliferation and differentiation into cartilage [9, 10]. The bFGF-stimulated cartilage-proliferation rabbit model in this study showed that perichondrial cells, rather than chondrocytes, were more likely to proliferate and differentiate into cartilage. Based on the proliferation activity of perichondrial cells analyzed by Ki67 labeling, it was suggested that the perichondrial cells in the outer layer of the perichondrial inner-layer/outer-layer boundary may have been involved in the observed proliferation. The perichondrium is composed of two layers: an outer layer in which small fibrocyte-like cells are interspersed between histologically sparse collagenous fibers, and an inner layer in which somewhat rounded cells in compact fibers have an irregular layer structure composed of three to four layers [9]. Kobayashi et al. demonstrated by immunostaining for the auricular-perichondrial MSC markers CD44 and CD90 in an in vitro study using human auricular perichondrial cells that in the inactive perichondrium, MSCs exist as elongated spindle-shaped cells located at the inner layer-layer/outer-layer boundary [2]. It has been shown that CD44-positive and CD90-positive MSCs in the perichondrial inner-layer/outer-layer boundary are activated by bFGF, become active chondrocyte precursor cells with short spindle shapes to star-like shapes, and participate in proliferation and chondrocyte differentiation [2]. It has also been shown that MSCs in the proliferated cartilage membrane are able to maintain long-term morphology by reforming the perichondrial membrane, with one of the roles of MSCs in the regeneration process of the perichondrium having been elucidated in this in vivo study.

### Verifying the effect of chondrogenesis following bFGF administration

**Fig. 5:**
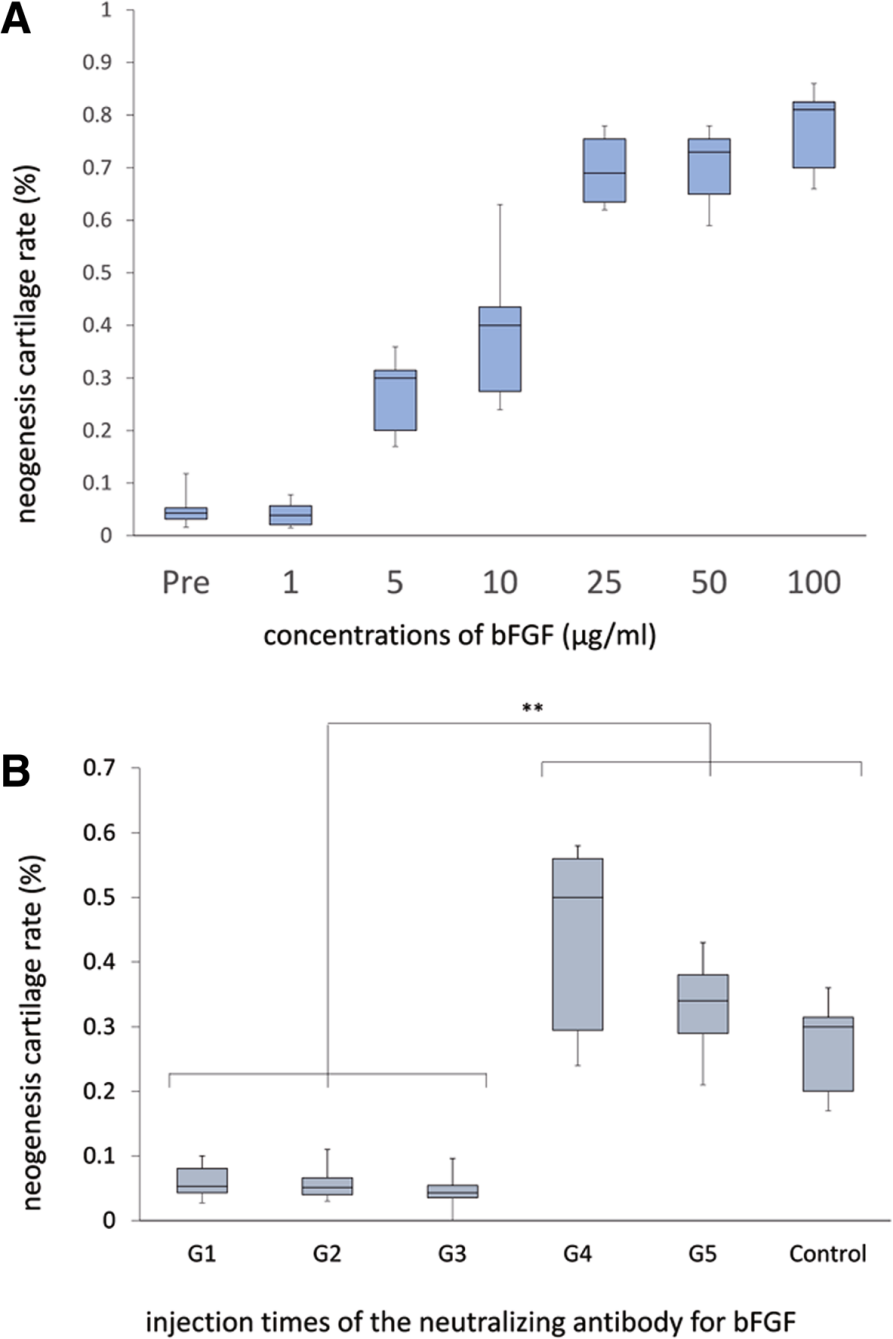
Evaluation of the effect of bFGF administration on cartilage proliferation. **a**. Concentration-dependent effect of bFGF on auricular chondrogenesis. The box plot shows the cartilage neogenesis rate for different concentrations of bFGF. Kruskal-Wallis analysis revealed a highly significant difference among the groups (*P* < 0.0001). Post hoc comparisons by the Fisher test indicated significant differences between the groups injected with more than 5 μg/ml and the control group, but the difference between animals injected with 1 μg/ml bFGF and the control group was not statistically significant. **b**. Blocking of bFGF stimulation using an anti-bFGF neutralizing antibody. The box plot shows the cartilage neogenesis rate immediately after injection of bFGF, immediately after and 1 week after injection of bFGF, immediately after and 2 weeks after injection of bFGF, 1 week after injection of bFGF, 2 weeks after injection of bFGF, and the control. Kruskal Wallis analysis revealed a significant difference between the three groups immediately after injection of bFGF and the control group (*P* < 0.01), but suppression of chondrogenesis at 1 week and 2 weeks after bFGF injection was not recognized in comparison with the control group. **P < 0.01. “Reprinted with permission from (Koizumi, N. (2010). Basic fibroblast growth factor stimulates proliferation and differentiation of perichondrocytes of rabbit auricular cartilage in vivo. Journal of Kanazawa Medical University, 35, 122–130.). Copyright (2010) publication administration of Kanazawa Medical University”

The effect of bFGF concentration on auricular chondrogenesis was shown in Fig. 5a. The average neogenesis cartilage rates were 0.046 ± 0.025 (control with no bFGF), 0.040 ± 0.020 (1 μg/ml bFGF), 0.28 ± 0.064 (5 μg/ml), 0.38 ± 0.11 (10 μg/ml), 0.70 ± 0.06 (25 μg/ml), 0.71 ± 0.061 (50 μg/ml), and 0.78 ± 0.07 (100 μg/ml). Kruskal-Wallis analysis revealed a highly significant difference among the groups (*P* < 0.0001). Post hoc comparisons by Fisher test indicated significant differences between the treatment groups that received more than 5 μg/ml of bFGF and the control group. However, the difference between the group that received 1 μg/ml bFGF and the control group was not statistically significant (*P* = 0.51).

The blockage of bFGF-induced chondrogenesis with an anti-bFGF neutralizing antibody was shown in Fig. 5b. The average cartilage neogenesis rates based on the ratio of new cartilage to total cartilage were 0.061 ± 0.024 in G1 (anti-bFGF neutralizing antibody injected immediately after bFGF injection), 0.055 ± 0.021 in G2 (anti-bFGF neutralizing antibody injected immediately after bFGF injection and at 1 week post injection), 0.046 ± 0.019 in G3 (anti-bFGF neutralizing antibody injected immediately after bFGF injection and at 2 weeks post injection), 0.44 ± 0.13 in G4 (anti-bFGF neutralizing antibody injected at 1 week post-bFGF injection), 0.33 ± 0.07 in G5 (anti-bFGF neutralizing antibody injected at 2 weeks post-bFGF injection), and 0.27 ± 0.06 for controls not receiving any anti-bFGF neutralizing antibody injections after the bFGF injection. Kruskal-Wallis analysis revealed a significant difference among the three groups immediately after injection of bFGF (G1–3) and the control group (*P* < 0.01), but suppression of chondrogenesis at 1 week and 2 weeks after bFGF injection was not recognized in comparison with the control group. The difference in the neogenesis rates between G1, G2, and G3, and between G4 and G5 were not significant.

The results from the current study confirmed that bFGF-induced chondrogenesis was concentration-dependent, with the highest concentration tested of 100 μg/ml yielding the highest levels of cartilage formation. It has been reported in vitro that the bFGF concentration at which chondrocytes show proliferation-promoting activity is 10 ng/ml [11, 12]. In our study, the concentration of bFGF that induced the highest degree of chondrogenesis was the same as that used in clinical practice for intractable ulcers and burn ulcers, and appeared to be a reasonable concentration for cell stimulation in vivo. The results from the experiments using anti-bFGF neutralizing antibodies revealed that chondrogenesis was inhibited in the groups that received the neutralizing antibody immediately after bFGF treatment, whereas in the group that received the neutralizing antibody at 1 week and 2 weeks post bFGF-injection, cartilage formation was not inhibited. Therefore, the process by which injected bFGF binds to and releases proteoglycans in vivo and the possibility that bFGF from cells in the perichondrial region is continuously produced is unlikely. The concentration-dependent bFGF-induced cartilage proliferation and suppression of cartilage proliferation by anti-bFGF neutralizing antibodies suggests that the stimulation of perichondrial cells by the injection of high concentrations of bFGF may have turned on a switch that promoted sustained proliferation of tissue stem cells or perichondrial progenitor cells present in the perichondrium, as well as their differentiation into chondrocytes. Nonetheless, it is unlikely that the initial time lag for significant proliferation of CD44-positive and CD90-positive MSCs or cartilage precursor cells of the perichondrium that was observed after bFGF stimulation was due to the direct growth-stimulating effect of bFGF on MSCs. We speculated that this effect was caused by angiogenesis induced by bFGF. Therefore, we examined the effects of an MMP inhibitor that inhibits angiogenesis, and anti-VEGF neutralizing antibodies.

### Verifying the inhibition of chondrogenesis through the inhibition of angiogenesis

**Fig. 6:**
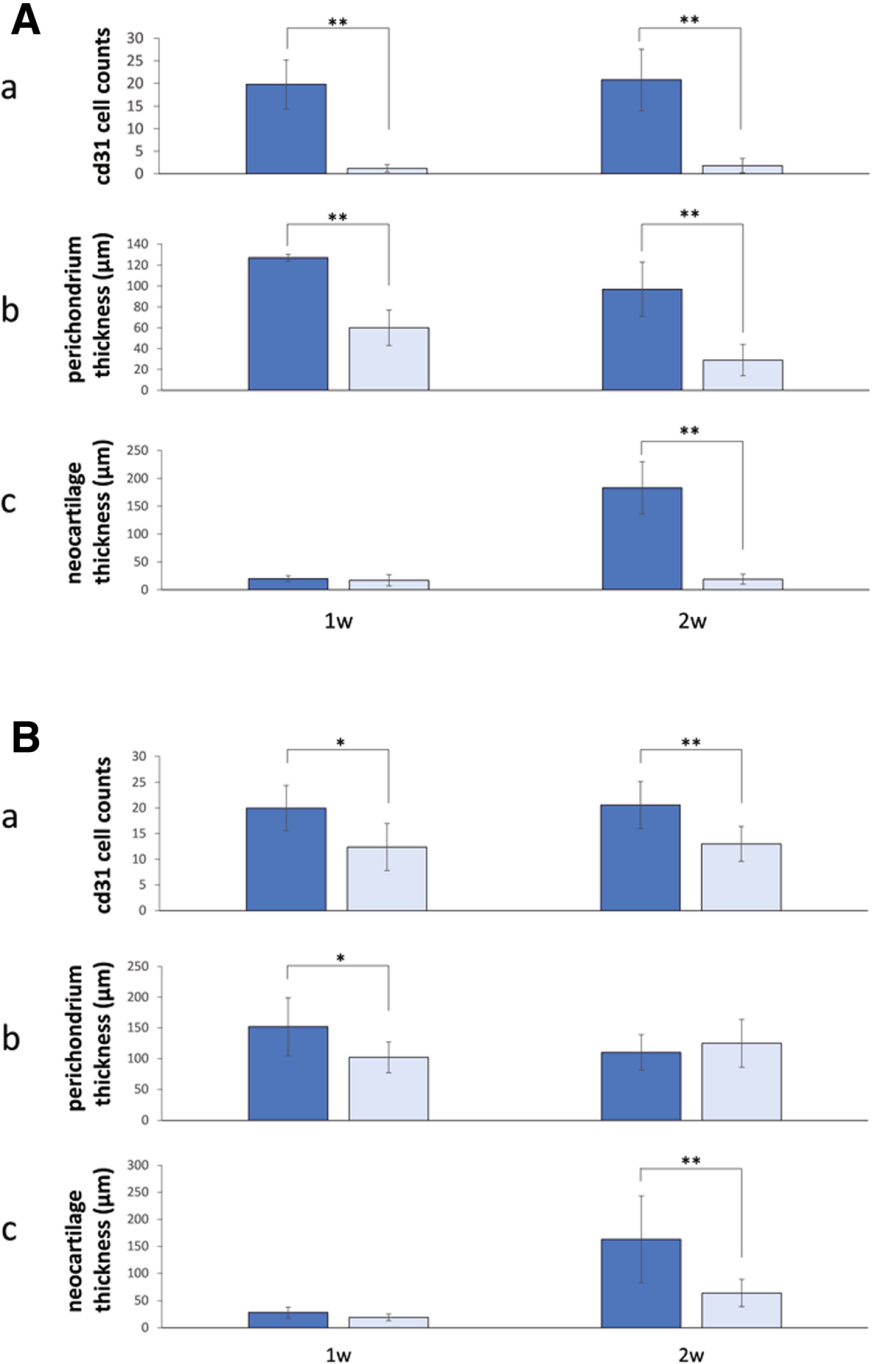
Cartilage proliferation stimulated by bFGF-induced vascularization. **a**. MMP inhibition. (a) Quantification of CD31-positive cells. Immunohistochemical staining with the anti-CD31 antibody was performed to evaluate angiogenesis. The graph shows the mean numbers of CD31-positive cells per visual field in the control groups (DMSO injection) and the experimental groups (MMP inhibitor injection). The differences in the numbers of CD31-positive cells between the corresponding control and experiment groups were statistically significant. **P < 0.001. (b) Perichondrium thickness. The graph shows the mean thickness of the perichondrium per microscopic visual field in the control groups (DMSO injection) and the experimental groups (MMP inhibitor injection). The differences between the corresponding control and experiment groups were statistically significant. **P < 0.01. (c) Neocartilage thickness. The graph shows the mean thickness of new cartilage per microscopic visual field in the control groups (DMSO injection) and the experimental groups (MMP inhibitor injection). The differences between the control and experimental groups at 2 weeks post bFGF-treatment showed statistically significant improvement in the experimental groups, but improvement was not observed at 1 week post treatment. **P < 0.001. **b**. VEGF neutralization. (a) Cell counts (CD31). The graph shows the mean numbers of CD31-positive cells per visual field in the control groups (DMSO injection) and the experimental groups (VEGF-neutralizing antibody injection). The differences in the counts of CD31-positive cells between the corresponding control and experiment groups were statistically significant. *P < 0.05; **P < 0.01. (b) Perichondrium thickness. The graph shows the mean thickness of the perichondrium per microscopic visual field in the control groups (DMSO injection) and the experimental groups (VEGF-neutralizing antibody injection). The differences between the control and experimental groups at 1 week post bFGF-treatment were statistically significant, but a significant difference was not observed at 2 weeks post treatment. *P < 0.05. (c) Thickness of neocartilage. The graph shows the mean thickness of new cartilage per microscopic visual field in the control groups (DMSO injection) and the experimental groups (VEGF-neutralizing antibody injection). The differences between the control and experimental groups at 2 weeks post bFGF-treatment were statistically significant, but no significant difference was observed at 1 week post treatment. **P < 0.001. “Reprinted with permission from (Miyanaga, A. (2017). Transient vascularization promotes proliferation and cartilage-formation of perichondrial progenitor cells. Journal of Kanazawa Medical University, 42, 24–31.). Copyright (2017) publication administration of Kanazawa Medical University”

The CD31 positive cells, the perichondrium and neocartilage thickness by administration of MMP inhabitation are shown in Fig. 6a. Immunohistochemical staining with the anti-CD31 antibody was performed to evaluate angiogenesis (Fig. 6a, graph panel a). The average numbers of positive cells per visual field in the control groups that received only an injection of the carrier DMSO were 19.8 ± 5.4 at 1 week and 20.8 ± 6.8 at 2 weeks, compared to 1.2 ± 0.8 at 1 week and 1.8 ± 1.6 at 2 weeks in the experimental groups that received the injections of a monoclonal antibody inhibiting MMP. The differences in the numbers of CD31-positive cells between the controls and the corresponding experimental groups were statistically significant (*P* < 0.001 at 1 week and < 0.001 at 2 weeks post treatment). The average perichondrium thickness per microscopic visual field in the control group was 127 ± 3 μm at 1 week, and 92 ± 26 μm at 2 weeks compared to 60 ± 17 μm at 1 week and 29 ± 15 μm at 2 weeks in the experimental group (Fig. 6a, graph panel b). The differences between the controls and the corresponding experimental groups were statistically significant (*P* < 0.01 at 1 week and < 0.01 at 2 weeks). The average neocartilage thickness per visual field in the control group was 20 ± 5 μm at 1 week and 183 ± 47 μm at 2 weeks, compared to 17 ± 10 μm at 1 week and 19 ± 9 μm at 2 weeks per field in the experimental groups (Fig. 6a, graph panel c). The differences between the controls and the experimental groups at 2 weeks post-treatment were statistically significant (*P* < 0.001). This differed from the results obtained at 1 week post-treatment, in which there was no significant difference between the control and experimental groups.

The CD31 positive cells, the perichondrium and neocartilage thickness by administration of VEGF neutralization are shown in Fig. 6b. The average number of positive cells per visual field in the control group that received injections of only the carrier DMSO was 20.0 ± 4.4 at 1 week and 20.6 ± 4.6 at 2 weeks post treatment, compared to 12.4 ± 4.6 at 1 week and 13 ± 3.4 at 2 weeks post treatment in the experimental groups (Fig. 6b, graph panel a). The differences in the numbers of CD31-positive cells between the controls and the corresponding experimental groups were statistically significant (*P* < 0.05 at 1 week and < 0.01 at 2 weeks post treatment). The average perichondrium thickness per visual field in the control groups was 152 ± 47 μm at 1 week and 110 ± 29 μm at 2 weeks post treatment, compared to 102 ± 25 μm at 1 week and 125 ± 39 μm at 2 weeks post treatment in the experimental groups (Fig. 6b, graph panel b). The differences between the control groups and the corresponding experimental groups at 1 week post-treatment were statistically significant (*P* < 0.05), but this did not continue, as no significant difference was detected at 2 weeks post treatment. The average neocartilage thickness per visual field in the control group was 28 ± 10 μm at 1 week and 163 ± 80 μm at 2 weeks post-treatment, whereas in the experimental groups it was 19 ± 6 μm at 1 week and 64 ± 25 μm at 2 weeks post treatment (Fig. 6b, graph panel c). The differences between the controls and the experimental groups at 2 weeks post-treatment were statistically significant (*P* < 0.001), but not at 1 week post-treatment where no significant difference was observed. Angiogenesis induced by bFGF was sensitive to an angiogenesis inhibitor, which suppressed bFGF-induced perichondrium proliferation and neocartilage formation.

The results from the MMP inhibitor and VEGF neutralization experiments showed that the proliferation of new blood vessels 1 week after bFGF stimulation and the proliferation of perichondrial cells 1 week after bFGF stimulation were both inhibited, similarly to cartilage formation 2 weeks after treatment. Therefore, it appears that new blood vessels were strongly involved in perichondrial proliferation and subsequent cartilage formation. Humanized anti-VEGF monoclonal antibody (common name, bevacizumab) utilizes VEGF as a target molecule and inhibits angiogenesis [13, 14]. It has also been reported that mononuclear cells express and secrete IL-1β, and induce VEGF genes of the vascular endothelium and fibroblasts [15]. Because mononuclear cells accumulated in the perichondrial region 1 day after the administration of bFGF, it is possible that bFGF induced early-stage mononuclear cell infiltration that stimulated VEGF, which in turn may have been involved in the observed cartilage proliferation. In addition, the MMP inhibitor batimastat (chemical name) inhibits MT1-MMP, MMP-2, MMP-9, and angiogenesis [16], apparently resulting in inhibition of cartilage proliferation. Additionally, the MMP inhibitor suppressed the enzymatic activity of MMP-1, which degrades type I collagen, the main extracellular-matrix component of the perichondrium [17]. Based on the results of our study, the reason for the observed strong inhibition of perichondrial proliferation and cartilage formation by the MMP inhibitor compared to the VEGF-neutralizing antibody may have been not only the inhibition of angiogenesis, but also the inhibition of invasion of MMP-1-positive cells on days 1 to 3 post bFGF-treatment. Takebe et al. found that early interactions with endothelial cells in establishing avascular tissues from human specific progenitors trigger the initial expansion of cartilage progenitor cells and promote the self-aggregation of a 3D condensation of progenitors without any scaffold materials in vitro, and the introduction of MSCs into immunodeficient mice results in angiogenesis within 3 days of grafting [4]. They also found that cartilage precursor cells proliferated from day 2 to 7 post-grafting, and that the grafted cells differentiated into chondrocytes from days 10 to 20 [4]. This is similar to the gradual changes observed in the bFGF-stimulated cartilage proliferation model analyzed in our study. They also reported that the onset of angiogenesis during the early stage of grafting is consistent with the timing of proliferation of MSCs. In fact, blocking of angiogenesis strongly inhibits the proliferation of cartilage progenitor cells and cartilage formation, and angiogenesis is essential for the proliferation of MSCs (cartilage precursor cells) derived from the perichondrium and their differentiation into chondrocytes [4]. In the current study, the timing of angiogenesis and that of proliferation of MSCs were consistent, whereas cartilage formation and the proliferation of the cartilage membrane were suppressed as a result of angiogenesis inhibition. Therefore, it appears that MSCs were activated in vivo by angiogenesis induced by the administration of bFGF and that the activated MSCs caused perichondrial proliferation and cartilage formation.

## Conclusions

The results of this study suggest that angiogenesis may be important for the induction of activation and differentiation of MSCs/cartilage precursor cells in vivo, and the maintenance of long-term tissue morphology. In the future, we hope to establish a novel reconstruction method for auricular cartilage using perichondrial MSCs, and clarify the details of the mechanisms involved.

## Abbreviations

MSC: Mesenchymal stem cell
bFGF: Basic fibroblast growth factor
MMP1: Matrix metalloproteinase 1
HE: Hematoxylin and eosin
DAB: 3,3′-diaminobenzidine
ANOVA: Analysis of variance
